# Synchronizing brains and hearts: A practical guide for caregiver–child fNIRS-ECG multimodal hyperscanning

**DOI:** 10.3758/s13428-026-03060-7

**Published:** 2026-06-17

**Authors:** Yelim Hong, Nicole J. Moore, Laura E. Quiñones-Camacho

**Affiliations:** Department of Educational Psychology, The University of Texas, 1912 SpeedwaySZB 5.708, Austin, TX 78712 USA

**Keywords:** Syncyrony, Multimodal hyperscanning, Functional near-infrared spectroscopy (fNIRS), Electrocardiography (ECG)

## Abstract

The study of synchrony—concordant neural, physiological, and behavioral activity between individuals—has advanced our understanding of co-regulation and socio-emotional development, particularly within caregiver–child relationships. While previous research has often examined neural and physiological synchrony separately, recent multimodal approaches highlight the importance of integrating these systems. This paper presents a comprehensive, practical guide to multimodal hyperscanning using functional near-infrared spectroscopy (fNIRS) and electrocardiography (ECG) in caregiver–child dyads. We outline theoretical foundations, methodological considerations, and detailed protocols for synchronizing fNIRS (via NIRx), ECG (via Mindware), behavioral video (via Mangold VideoSyncPro), and task stimuli (via PsychoPy) in a fully time-aligned manner. We describe equipment configurations, software integration, trigger-based synchronization workflows, and strategies to overcome common challenges in developmental psychophysiological research, particularly with young children. Drawing from our experience with children aged 3–7 years, we provide empirical benchmarks for feasibility, compliance, and data quality. This guide provides a practical, adaptable framework designed to lower the barrier to entry for multimodal hyperscanning and encourage its widespread use in developmental research. Integrating neural and physiological synchrony allows researchers to capture the dynamic, multi-systemic nature of caregiver–child interaction and opens new avenues for investigating mechanisms of co-regulation, emotional development, and risk for psychopathology.

**Supplementary Information:**

The online version contains supplementary material available at 10.3758/s13428-026-03060-7.

## Introduction

### What is synchrony? Why is it important?

The study of caregiver–child synchrony – the concordant neural, physiological, and behavioral activity observable in caregiver–child interactions – has gained popularity in the past few decades (Davis et al., 2017; Harrist & Waugh, 2002; Leclère et al., 2014; Quiñones-Camacho et al., 2020; Quiñones-Camacho et al., 2022). Interest in the study of synchrony comes, in part, because of how influential caregiver–child synchrony is for the development of social and affective systems (Feldman, 2007a, 2007b). Moreover, synchrony has been observed across social contexts, including caregiver–child dynamics, romantic couples, and friendships, and has been examined across multiple modalities such as behavioral, physiological, and neural activity (Davis et al., 2017; Feldman, 2007a, 2007b; Mayo & Gordon, 2020). Among these, caregiver–child synchrony has become popularized as a key variable of developmental study, demonstrating relevance across domains such as self-regulation (Feldman et al., 1999) and psychopathology (Quiñones-Camacho et al., 2022) across childhood and adolescence.

While substantial work has focused on understanding behavioral forms of synchrony, the past two decades have highlighted the role of neural synchrony as a potential correlate of key developmental milestones such as caregiver–child coordination, attachment, communication, and psychopathology (Nguyen et al., 2020; Atzil & Gendron, 2017; Feldman, 2017; Quiñones-Camacho et al., 2022). At the same time, the interpretation of neural synchrony remains an active area of debate (Holroyd, 2022). Recent work has emphasized that claims about its developmental significance should be made cautiously and with attention to the neuroimaging modality and analytic approach used, particularly in fNIRS versus EEG hyperscanning (Roche et al., 2025), and also highlighted the need for longitudinal studies to clarify its contribution to developmental outcomes (Hoehl et al., 2025). These ongoing questions further underscore the importance of temporally aligned multimodal work proposed here that can clarify how neural, physiological, and behavioral synchrony co-occur and unfold over time.

In addition, while neural synchrony and peripheral synchrony (e.g., heart rate coordination) have traditionally been examined separately, recent studies have begun to explore their interaction and investigate how these processes are interconnected (Nguyen et al., 2021a, 2021b, 2021c; Palumbo et al., 2017; Stuldreher et al., 2019; Vanutelli et al., 2017). To advance this work, researchers have adapted methods originally developed for comparing various peripheral physiological signals or neural activity channels, creating composite measures that integrate neural and physiological data (Montague et al., 2002; Stratford et al., 2012; Stuldreher et al., 2019). This multimodal approach enhances the ecological validity of synchrony research, offering new opportunities to examine how psychological states are reflected in, and shaped by, dynamic physiological and neural processes across social contexts.

Behavioral synchrony represents another key level of alignment between partners. Behavioral synchrony, reflected in the temporal coordination of gaze, gestures, facial expressions, and vocal patterns, has been shown to support social bonding as well as cognitive and regulatory processes (Beebe et al., 2024; Feldman, 2012; Leclère et al., 2014). Because these behavioral dynamics often co-occur with and can predict physiological and neural coupling, multimodal studies should integrate behavioral synchrony to capture the full spectrum of dyadic coregulation. Although some work has combined behavioral data with neural or physiological measures (e.g., Busuito et al., 2019; Woltering et al., 2015), studies that simultaneously integrate multiple biological modalities with behavior remain rare. By integrating multiple biological systems with fine-grained behavioral data and synchronizing all modalities within a shared temporal framework, our approach helps address limitations of studies that rely solely on global or summary-level behavioral measures. We view behavioral synchrony as a foundational and complementary modality within this multimodal system. As such, the framework we present also incorporates synchronizing video recordings with the neural and physiological measures. This approach allows for assessing real-time overlap between behavior and both neurophysiological measures to get a more complete understanding of the caregiver–child relationship.

### Hyperscanning: A tool for investigating neural synchrony

Hyperscanning, the simultaneous measurement of neural activity in multiple individuals, has been utilized to study how the neural rhythms of multiple individuals become synchronized across varying environmental contexts and stimuli. Originally introduced in functional magnetic resonance imaging (fMRI) research by Montage and colleagues in 2002 (Montague et al., 2002), hyperscanning is now most commonly conducted with other neuroimaging technologies, such as functional near-infrared spectroscopy (fNIRS) and electroencephalogram (EEG). Hyperscanning is specifically fitting for the study of interpersonal, neural entrainment in contexts such as communication (Hasson et al., 2012), relationships (Montague et al., 2002), and emotional exchanges, as it allows for a mechanistic assessment of the underlying processes facilitating these interactions. As such, hyperscanning technology is an unrivaled method for studying the neural dynamics that underlie real-time social interactions, and specifically for investigating the role of neural synchronization in psychological development. This approach is especially valuable for exploring the underlying neural dynamics of caregiver–child social communication, socio-cognitive processes, and affective interactions, thereby advancing our understanding of developmental mechanisms and risk pathways of psychopathology from a social neuroscience perspective. Moreover, integrating hyperscanning with peripheral nervous system synchrony measures, like cardiac physiology, and real-time behavioral data allows for a holistic, multimodal perspective of how neural, physiological, and behavioral processes uniquely and interactively contribute to developmental processes.

### Advancing multimodal hyperscanning research

With an increasing number of hyperscanning studies and growing recognition of the importance of multimodal hyperscanning approaches, there is an increasing need for a streamlined guide to collecting multimodal (e.g., neural, peripheral, and behavioral synchrony) data. This includes ensuring high data quality, preserving participant comfort, and achieving seamless time-synchronization across data streams during experimental tasks. While a few studies have conducted multimodal hyperscanning, there remains a need for a standardized method for incorporating multiple streams of data into multimodal hyperscanning paradigms in a fully time-synchronized manner. Despite the growing body of research on hyperscanning, most existing paradigms have focused on a single-modality approach. In contrast, our approach highlights the benefits of concurrently collecting multimodal data, including neural, cardiac, and behavioral measures, within a fully time-synchronized framework.

We emphasize the importance of developing best practices for multimodal hyperscanning paradigms, particularly those aimed at uncovering the neural and physiological mechanisms underpinning caregiver–child interactions. Our methodology supports real-time, simultaneous data collection across modalities, enabling a more holistic examination of dynamic social processes such as co-regulation, emotional attunement, and behavioral coordination. The goal of the current paper is to introduce the details of our multimodal hyperscanning protocol, as well as the practical strategies we have developed that allow for this extensive data collection. We aim to demonstrate the feasibility and importance of combining fNIRS and ECG within a multimodal hyperscanning framework in caregiver–child dyads, providing novel insights into the biological correlates of dyadic social processes. Our aim is to provide a practical and replicable framework that other researchers can adopt, thereby broadening the scope of multimodal hyperscanning method research and advancing the field toward a standardized approach for fully synchronized, multimodal data collection.

## Methods for examining neural synchrony

### What methods have been used to examine neural synchrony?

Hyperscanning studies have used various neuroimaging techniques, such as fNIRS (Czeszumski et al., 2022), EEG (Goldstein et al., 2018), magnetoencephalography (MEG; Hirata et al., 2014), and fMRI (Koike et al., 2016). Each method and its corresponding equipment offer distinct benefits and limitations for hyperscanning applications (Ayrolles et al., 2021; Czeszumski et al., 2020). A large amount of caregiver–child hyperscanning research has relied on fNIRS, which monitors hemodynamic responses—that is, changes in blood flow and oxygenation in the brain associated with brain activity, or EEG, which records the electrical activity generated by groups of neurons. These techniques are particularly suited for studying naturalistic interactions with children, as they allow for face-to-face engagement and relatively unrestricted movement (Alonso et al., 2024).

### Why focus on fNIRS hyperscanning?

Although the number of adult hyperscanning studies has steadily increased, developmental hyperscanning research remains challenging. fNIRS is a promising tool for advancing this work. It uses a lightweight cap fitted with optodes (light sources and detectors) that emit near-infrared light to non-invasively and indirectly measure neural activity by tracking changes in blood oxygenation levels. The light penetrates the scalp, and neural activity in the outermost cortical layers is captured through specific channels formed between optodes in the probe set (Nguyen et al., 2021a, 2021b, 2021c). Similar to fMRI, fNIRS measures the hemodynamic response based on neurovascular coupling—the principle that increased neural activity results in increased blood flow and oxygenation (Lloyd-Fox et al., 2010). However, unlike fMRI, which relies on the blood-oxygenation-level-dependent (BOLD) signal—a measure of change in the ratio of oxygenated to deoxygenated blood, fNIRS provides more detailed information by separately measuring the relative concentrations of oxygenated hemoglobin (HbO), deoxygenated hemoglobin (HbR), and total hemoglobin (Hb). Typically, increased neural activity results in elevated HbO and total Hb levels, with a slight decrease in HbR (Nguyen et al., 2021a, 2021b, 2021c; Reindl et al., 2019).

fNIRS is particularly well suited for naturalistic developmental hyperscanning due to its tolerance for motion, portability, ease of use, and cost-effectiveness (Carollo et al., 2022; Reindl et al., 2019). Compared to EEG, which offers higher temporal resolution but is sensitive to motion artifacts, and fMRI, which provides high spatial resolution but limits movement, fNIRS offers a balanced approach between the advantages and limitations of both fMRI and EEG (Mehta & Parasuraman, 2013). It provides moderate spatial resolution, allows for real-time social interaction, and is less affected by participant movement, making it an ideal tool for studying live, dynamic social behavior (Carollo et al., 2022). Although fNIRS is a relatively newer technology compared to fMRI and EEG, its methodological approaches are rapidly evolving. However, further standardization is needed to enhance cross-study replicability (e.g., Bizzego et al., 2022b). Despite this, its adaptability has enabled researchers to design experimental paradigms that closely resemble everyday caregiver–child activities. For example, fNIRS hyperscanning has been used to examine mother–child brain synchrony during free play (Bizzego et al., 2022a) and has supported research in more naturalistic settings, such as caregiver–child interactions, educational settings, and musical environments (Carollo & Esposito, 2024). Existing fNIRS hyperscanning studies with children have demonstrated that caregiver–child dyads synchronize their brain activity during cooperative tasks, from standardized button-press tasks to more naturalistic interactions, when compared to control conditions (Nguyen et al., 2020; Quiñones-Camacho et al., 2020). Further research has also identified interpersonal neural synchrony between adults or caregivers and children during free play and while watching emotional videos (Piazza et al., 2020; Azhari et al., 2019).

The advent of fNIRS hyperscanning has opened new avenues for addressing critical developmental questions. Such as, what patterns of neural synchrony emerge during collaborative tasks in caregiver–child dyads? Also, can neural synchrony serve as a biomarker for caregiver–child relationship quality or early indicators of psychopathology? By enabling the measurement of brain activity in ecologically valid, interactive settings, fNIRS hyperscanning holds great promise for advancing developmental science and enriching our understanding of social and emotional development.

## Methods for examining peripheral physiological synchrony

### What methods have been used to examine peripheral physiological synchrony?

Several peripheral physiological signals have been used to assess interpersonal synchrony in dyadic research, particularly in the context of caregiver–child interaction, emotional coregulation, and stress transmission. Among these, electrocardiography (ECG) is the most widely used, as it provides reliable measures of heart rate and heart rate variability (e.g., RSA), which are closely linked to parasympathetic functioning and self-regulation capacity (Fuchs et al., 2021; Quiñones-Camacho & Davis, 2019; Zahn et al., 2016). In addition, pre-ejection period (PEP)—derived from ECG and impedance cardiography—indexes sympathetic nervous system activity and is often used to examine physiological engagement or effortful arousal (Albinet et al., 2024; Bush et al., 2011; e.g., DiGiovanni et al., 2024). Respiration has also been examined as a potential synchrony marker, as breathing rate and depth may synchronize between dyad members during emotionally evocative or calming tasks (e.g., Coutinho et al., 2025; Tschacher & Meier, 2020). In addition, skin conductance, which captures sympathetic arousal via sweat gland activity, has been used to assess co-arousal during emotionally intense or stressful contexts (Bach et al., 2010; Han et al., 2022; e.g., Rosebrock et al., 2016). While each of these measures provides unique insight into distinct branches of the autonomic nervous system and emotional functioning, ECG remains the most extensively validated and widely used peripheral physiological measure in developmental synchrony research, particularly due to its robustness, feasibility, and compatibility with behavioral and neural data streams (Marzoratti & Evans, 2022).

### Why focus on ECG synchrony?

Examining electrocardiogram (ECG) synchrony offers several advantages for understanding dyadic physiological coordination, particularly in caregiver–child interactions. One key strength of ECG synchrony is its high temporal precision compared to other autonomic nervous system measures, enabling the assessment of moment-to-moment physiological responses during emotionally and cognitively demanding tasks (Wang et al., 2025; Woody et al., 2016). This level of accuracy allows researchers to observe subtle fluctuations in autonomic nervous system activity, providing valuable insights into how dyads jointly regulate physiological states during real-time interaction. In developmental contexts, cardiac synchrony is particularly relevant for studying co-regulation, as it reflects the underlying physiological scaffolding that supports social and emotional development (Bornstein & Esposito, 2023; Coutinho et al., 2021; Shih et al., 2019).

One of the most commonly used measures of cardiac function is heart rate variability (HRV), derived from ECG recordings by calculating the average variation in time between heartbeats (i.e., interbeat interval or IBIs; Shaffer & Ginsberg, 2017). While HRV provides a general indicator of autonomic activation, it does not distinguish between the sympathetic and parasympathetic branches of the autonomic nervous system (Cacioppo et al., 1994; Thomas et al., 2019). To address this limitation, more specific measures of cardiac activity are often used to isolate distinct branches of the autonomic nervous system. Respiratory sinus arrhythmia (RSA) is one such measure, representing parasympathetic control of the heart via the vagus nerve (Cacioppo et al., 1994; Palumbo et al., 2017). RSA captures natural fluctuations in cardiac rhythm associated with respiration and is calculated by examining changes in interbeat interval (IBI) within the respiratory frequency range (e.g., 0.12–0.40 Hz; Cacioppo et al., 1994). By focusing on RSA, researchers can more precisely assess parasympathetic engagement and its role in emotion regulation and social connectedness.

Research has highlighted RSA synchrony as a significant dyadic process associated with children’s regulatory outcomes (Lunkenheimer et al., 2015). RSA is a reliable biomarker of self-regulation, as it reflects inhibitory parasympathetic control that slows heart rate and supports the maintenance of calm physiological states (Thayer & Lane, 2000). A calm physiological state, indicated by higher resting RSA, is thought to support enhanced cognitive functioning, adaptive social behaviors, and improved emotion regulation (Holzman & Bridgett, 2017; Patriquin et al., 2015; Porges, 2007). Conversely, lower resting RSA has been associated with increased psychological distress and difficulties in self-regulation in both children and adults (Beauchaine et al., 2019). Thus, RSA, which reflects individual differences in regulatory capacity and the propensity for social engagement (Holzman & Bridgett, 2017; Patriquin et al., 2015; Porges, 2007), may play a crucial role in shaping RSA synchrony.

ECG synchrony can become even more powerful when combined with fNIRS. While fNIRS captures neural synchrony in cortical regions associated with social and emotional processing (Carollo et al., 2022), ECG synchrony reflects fluctuations across the parasympathetic and sympathetic activity of the autonomic nervous system (Coutinho et al., 2021). Together, this multimodal approach offers a more comprehensive understanding of the interconnected neural and physiological mechanisms underlying dyadic interactions (Reindl et al., 2022). By combining ECG and fNIRS hyperscanning, we can address key research questions regarding how cardiac synchrony and neural synchrony converge during caregiver–child interactions. This approach enables researchers to explore how the autonomic nervous system and brain-based processes jointly support emotional co-regulation, a core function of close relationships (Reindl et al., 2022). Mounting evidence suggests that dyadic regulation is multi-systemic, involving interactions across neural synchrony (Quiñones-Camacho et al., 2022) and parasympathetic regulation (Lunkenheimer et al., 2015; Suveg et al., 2016). Investigating ECG synchrony alongside these systems can enhance our understanding of the biophysiological foundations of co-regulation and inform future research on caregiver–child interactions and developmental risk and resilience.

### Multimodal ECG-fNIRS hyperscanning: A new frontier?

While many researchers utilize fNIRS or ECG independently to study caregiver–child synchronization, few studies have included concurrent fNIRS and ECG data (Nguyen et al., 2021a, 2021b, 2021c, 2022; Reindl et al., 2022). In a paper by Nguyen and colleagues (2021), while fNIRS-based neural synchrony was related to the communicative signals during caregiver-infant physical touch, ECG-based physiological synchrony was related to co-regulatory affective signals. Further supporting these findings, Reindl and colleagues (2022) also found differential effects for neural and physiological synchrony during a competition and cooperation task. These findings suggest independent, yet co-regulatory functions of both types of biological synchrony. While these studies make a substantial contribution to the field of biological synchrony, they represent only a small fraction of the broader body of research on biological synchrony. The lack of ECG and fNIRS multimodal hyperscanning seems to be primarily driven by practical limitations such as increased risk of technical difficulties, the lack of wireless connectivity in some fNIRS systems, and potential participant discomfort. Adding another layer of complexity, time-synchronized multimodal hyperscanning requires additional equipment and resources that may not be available in all labs. Despite these constraints, time-synchronized concurrent ECG and fNIRS hyperscanning data offer a unique opportunity to disentangle bottom-up arousal processes from top-down neural processes and are therefore a significant contribution to the field of hyperscanning. To demonstrate the feasibility of such a setup and encourage broader implementation of similar setups, we describe in the following sections how we have successfully implemented this methodology in our laboratory.

## Experimental equipment

### fNIRS recording: NIRx

Selecting an appropriate system is critical for successful caregiver–child hyperscanning. We selected the NIRx platform, specifically the Nirsport 2 (NIRSport2®; NIRx Medical Technology, Berlin, Germany) devices, due to its technologically advanced features, strong signal quality, mobility, and flexibility for developmental and multimodal research. The Nirsport 2 offers a modular optode arrangement, with the base model including eight sources and eight detectors, and expandable up to 80 sources and 80 detectors. These optodes can be arranged in customizable montages to capture neural activity in different regions of the cerebral cortex. Nirsport caps come in various sizes, are lightweight (~ 900 g), making them suitable for tasks that involve regular bodily movement, and can be paired with other modalities, such as EEG (Nguyen et al., 2021a, 2021b, 2021c), eye trackers (da Silva Soares et al., 2022), or virtual reality (VR) systems (Landowska et al., 2018). To reduce the risk of mistaking extra-cerebral signals or bodily movement-induced noise for true cortical activity, the Nirsports offer a full array of short-distance detectors and an integrated accelerometer. These features are critical for isolating true cortical activity, as superficial signals (i.e., scalp perfusion, bodily movements, respiration, Meyer waves, cardiac signals, etc.) can emulate cortical hemodynamic responses. These short channels serve as a reference for superficial noise, which can be regressed out during preprocessing to improve the accuracy of cortical signal estimation (Gagnon et al., 2014).

During calibration, the software checks that sources emit light properly, detectors receive the signal, and no significant environmental interference (i.e., light pollution) is present. Poor signal may occur as a result of sub-optimal contact between the optodes and the skull, caused by wire or hair interference or light pollution in the environment. For the former, increasing the spring-top pressure or gently moving hair/wires out of the way typically improves the signal. In case of light pollution, placing a shower cap over the fNIRS cap can help block external light and reduce interference. After calibration, the system enables monitoring of oxygenated and deoxygenated hemoglobin concentrations at different temporal resolutions in real-time (Chen et al., 2020). Furthermore, NirX’s basic acquisition software (Aurora) can be integrated with task software, like PsychoPy (Peirce et al., 2019) for automated event marking (Mutlu et al., 2020). Event markers allow researchers to analyze neural responses in relation to stimulus onset, enhancing temporal alignment between stimulus presentation and the corresponding hemodynamic response (Sánchez-Reolid et al., 2023).

Data collected in Aurora are automatically saved in the SNIRF file format, which is compatible with open-source platforms such as Homer3 (Huppert et al., 2009) or NIRS Toolbox (Santosa et al., 2018). These platforms are freely available and widely used in the fNIRS research community. Although both offer graphical user interfaces (GUIs), they may still require greater familiarity with preprocessing workflows and analytic decisions than more automated platforms. Alternatively, data preprocessing can be conducted utilizing either Satori, the Nirx-specific analysis software, which provides a streamlined and user-friendly interface. However, it requires a separate purchase and is relatively expensive.

Anatomical differences across age groups, such as skull thickness, scalp-to-cortex distance, and head size, influence optimal optode spacing and cortical sensitivity profiles (Beauchamp et al., 2011). For infants, source–detector distances of 20–25 mm are recommended, while preschool-aged children typically require 25–30 mm spacing, and school-aged children and adults 30–35 mm (Aslin & Mehler, 2005). These adjustments ensure adequate cortical penetration and minimize cross-talk between superficial and cortical signals. To account for developmental anatomical variability in cortical mapping, we recommend using devFOLD (Developmental fNIRS optodes location decider) application (Fu & Richards, 2021). devFOLD extends the original fOLD toolbox (Zimeo et al., 2018) by incorporating age-appropriate MRI head models for infants, children, and adults. This allows researchers to design probe geometries and estimate channel-to-region of interest (ROI) sensitivity using developmental anatomical templates, ensuring that optode placements are appropriate for each age group. By estimating cortical sensitivity and channel registration based on developmental head models, devFOLD helps ensure that optode positions reliably capture the intended cortical regions across ages (Fu & Richards, 2021). Incorporating tools such as devFOLD can enhance cross-age comparability and the validity of developmental hyperscanning data. A more detailed explanation of NIRx equipment is presented in Appendix B.

#### Considerations for system selection

When selecting a fNIRS system for hyperscanning applications, researchers should first identify the specific aims and practical constraints of their study. Critical factors include the target population (e.g., infants, children, adults) and corresponding cap fit and comfort, as well as the availability of appropriately sized headgear to minimize motion artifacts and ensure participant compliance. Hardware modularity is another major consideration, as systems vary in whether they allow full-head, only forehead, or customizable montages. Researchers should evaluate the availability of short-separation channels, which are critical for separating cortical from superficial signals. Another consideration includes setup and calibration time, which affects feasibility in studies involving young children or field-based data collection, and the number of devices that can be recorded simultaneously for hyperscanning designs. In addition, the degree of wireless capability and data transmission stability (e.g., Wi-Fi vs. wired) influences participant movement flexibility and recording stability. Finally, multimodal compatibility (e.g., with ECG, EEG, or eye-tracking) and accessible synchronization methods are critical for integrated multimodal designs. By focusing on these criteria, researchers can choose systems that best meet their needs even as technologies evolve or individual companies change over time. We provide example companies that currently offer systems well suited for hyperscanning and developmental research in Appendix C.

### ECG recording: MindWare

Selecting an appropriate ECG platform for concurrent dual recording is essential for capturing high-quality physiological data in caregiver–child research. Mindware Technologies offers a widely used mobile ECG system well-suitable for collecting cardiac and respiratory activity in multimodal hyperscanning designs (Kleckner et al., 2021). MindWare’s (MindWare Technology, Gahanna, OH, USA) acquisition software, BioLab, captures and displays real-time ECG and respiration data from multiple individuals simultaneously. Moreover, it offers customizable scaling and filtering options to optimize signal quality and supports integration with other platforms for synchronized multimodal data collection (Bush et al., 2016). For example, BioLab can be synchronized with Mangold’s VideoSyncPro (a video recording platform described in detail below) to achieve frame-level precision in aligning physiological and behavioral events. MindWare’s analysis software (HRV and IMP) can also be integrated with behavioral coding systems such as Mangold INTERACT (Richardson et al., 2019). In addition, BioLab can be further integrated with PsychoPy, a third-party experimental software, allowing for precise synchronization of physiological signals with stimulus presentation. Because behaviors and physiological responses happen quickly, having fully integrated acquisition and analysis systems enhances the accuracy and interpretability of observed associations between behavioral cues and underlying physiological processes.

Collecting ECG and RSA data across development requires consideration of age-related physiological and anatomical differences. Compared to adults, children exhibit higher and more variable respiratory rates, smaller thoracic volume, and thinner skin, all of which affect electrode placement and frequency parameters used in RSA estimation. ECG data should be sampled at least 500 Hz to ensure accurate R-peak detection, and preprocessing should include carefully selected high-pass and low-pass filters to preserve cardiac signals while minimizing noise. RSA, reflecting high-frequency heart rate variability linked to respiration, should be analyzed using an age-appropriate band-pass filter corresponding to typical respiratory frequencies (e.g., 0.24–1.04 Hz for children;.12–.40 Hz for adults; Shaffer & Ginsberg, 2017; Lackner et al., 2020). As respiration rates decrease with age, the optimal high-frequency band shifts accordingly (Shader et al., 2018). Because young participants are more prone to movement and electrode displacement artifacts, semi-automated R-peak editing combined with visual inspection is especially recommended. Implementing these age-sensitive procedures ensures physiologically valid measurement of RSA and related autonomic indices, supporting reliable comparisons across developmental stages in dyadic and hyperscanning research. A more detailed explanation of Mindware equipment is presented in Appendix B.

#### Considerations for system selection

In planning to collect ECG data for developmental and/or hyperscanning research, researchers should consider both their methodological goals and the physiological characteristics of their participants. Key considerations include the target population (e.g., infants, children, adults) and the availability of appropriately sized and comfortable electrodes, which help ensure participant compliance and minimize noise caused by movement or poor contact. For developmental samples in particular, additional attention is needed to ensure proper electrode fit, placement stability, and comfort, as children tend to be more sensitive to adhesives and more prone to movement, which can affect data quality. Signal quality and sampling rate are also essential for accurate R-peak detection and RSA estimation, with systems supporting sampling frequencies of at least 500 Hz. The inclusion of features that allow measurement of additional autonomic indices (e.g., PEP, CO, SV) can further expand analytic scope. Researchers should also evaluate setup time, device portability, and battery life, as these factors influence feasibility in studies with young children and in naturalistic contexts or field-based contexts. The number of concurrent recordings and channel flexibility determine whether the system can accommodate dyadic, group-based, or multimodal designs, while integration and synchronization capabilities with behavioral coding platforms or neural modalities (e.g., fNIRS) enhance temporal precision. Selecting an ECG system based on these generalized criteria, researchers can ensure high-quality, developmentally appropriate physiological data that remain attainable even as specific technologies evolve. We provide examples of alternative companies offering EEG systems in Appendix C.

### Video recording: Mangold international

The choice of software for behavioral coding and video synchronization is crucial, as each platform offers distinct strengths tailored to specific research needs. Mangold International, through its VideoSyncPro Studio and Mangold INTERACT (Mangold, 2018), provides a robust, user-friendly platform for synchronized video-based behavioral coding with options for physiological data integration (Grassl, 2020). It stands out for its multimodal integration capabilities, flexibility, and comprehensive analytical possibilities. Developed for behavioral research, it provides high-precision, frame-accurate synchronization, and includes an intuitive interface for coding behaviors from multiple individuals in the same video (Mangold Interact), making it well-suited for complex observational studies and analyses for behavioral synchrony. A more detailed explanation of Mangold software is presented in Appendix B.

#### Considerations for system selection

The choice of platform should be guided by the specific priorities and methodological needs of the research project—whether the focus is on behavioral coding, physiological data integration, motion tracking, or cloud-based collaboration. For studies examining multimodal synchrony, accurate alignment between behavioral events and physiological signals (e.g., ECG, fNIRS) is essential. Systems should facilitate reliable synchronization of event markers across platforms and allow timestamps to be easily imported into analysis software for multimodal alignment. Researchers should also consider the resolution, frame rate, and audio quality needed to capture behavioral cues of interest, as these parameters influence the precision and interpretability of behavioral coding. While interface design and usability remain relevant, the primary consideration is the system’s capacity to maintain consistent temporal correspondence across data streams. Choosing a setup with strong integration features ensures accurate and interpretable behavioral–physiological synchrony data. We provide examples of alternative companies offering behavioral coding and multimodal observation systems in Appendix C.

### Task software: PsychoPy

PsychoPy is a widely used, open-source software library that allows researchers to create a wide range of visual and auditory stimuli and design flexible experimental paradigms within a Python-based scripting framework. As a freely available tool, it offers extensive customization options and supports integration with various external hardware systems (e.g., fMRI, MEG, and a range of biometric sensors), making it particularly well-suited for psychophysiological and neuroimaging research (Peirce, 2009). Its compatibility with diverse devices also makes it valuable for multimodal experiments. Using standard Python syntax, researchers can easily design and customize experiments, including the integration of real-time physiological data collection and custom event triggers tailored to specific research questions. In addition, PsychoPy plays a crucial role in event triggering and synchronization with physiological recording systems, such as ECG, fNIRS, and EEG. It allows researchers to send real-time event markers, ensuring precise temporal alignment between stimulus onset and physiological data acquisition (Peirce, 2009). This level of synchronization is essential for studies investigating neural and physiological responses to cognitive and emotional stimuli, where accurate timing between stimulus presentation and physiological responses is critical.

#### Considerations for system selection

Selecting task software for multimodal hyperscanning research requires consideration of its ability to achieve precise timing and integration across recording modalities. Researchers should evaluate how well a platform supports the synchronization of event markers with physiological acquisition software (e.g., fNIRS, ECG) and whether triggers can be transmitted reliably and concurrently across devices. Additional factors such as timing accuracy, stability, and reproducibility across trials are essential for studies examining dynamic physiological responses. Choosing a task platform according to these generalized criteria ensures valid temporal correspondence between stimulus presentation and physiological reactivity across modalities. We provide examples of alternative companies offering task software systems in Appendix C.

## Synchronization of fNIRS, ECG, PsychoPy, and Mangold VideoSyncPro

To ensure precise synchronization across multiple data acquisition systems, we implemented a structured approach using software-based event markers, hardware triggers, and a shared port replicator. This integrated setup aligns fNIRS (NIRx Aurora/Hyperscan), ECG (MindWare BioLab), task stimuli (PsychoPy), and video recordings (Mangold VideoSyncPro) to a common temporal reference, enabling accurate multimodal data analysis.

**Fig. 1:**
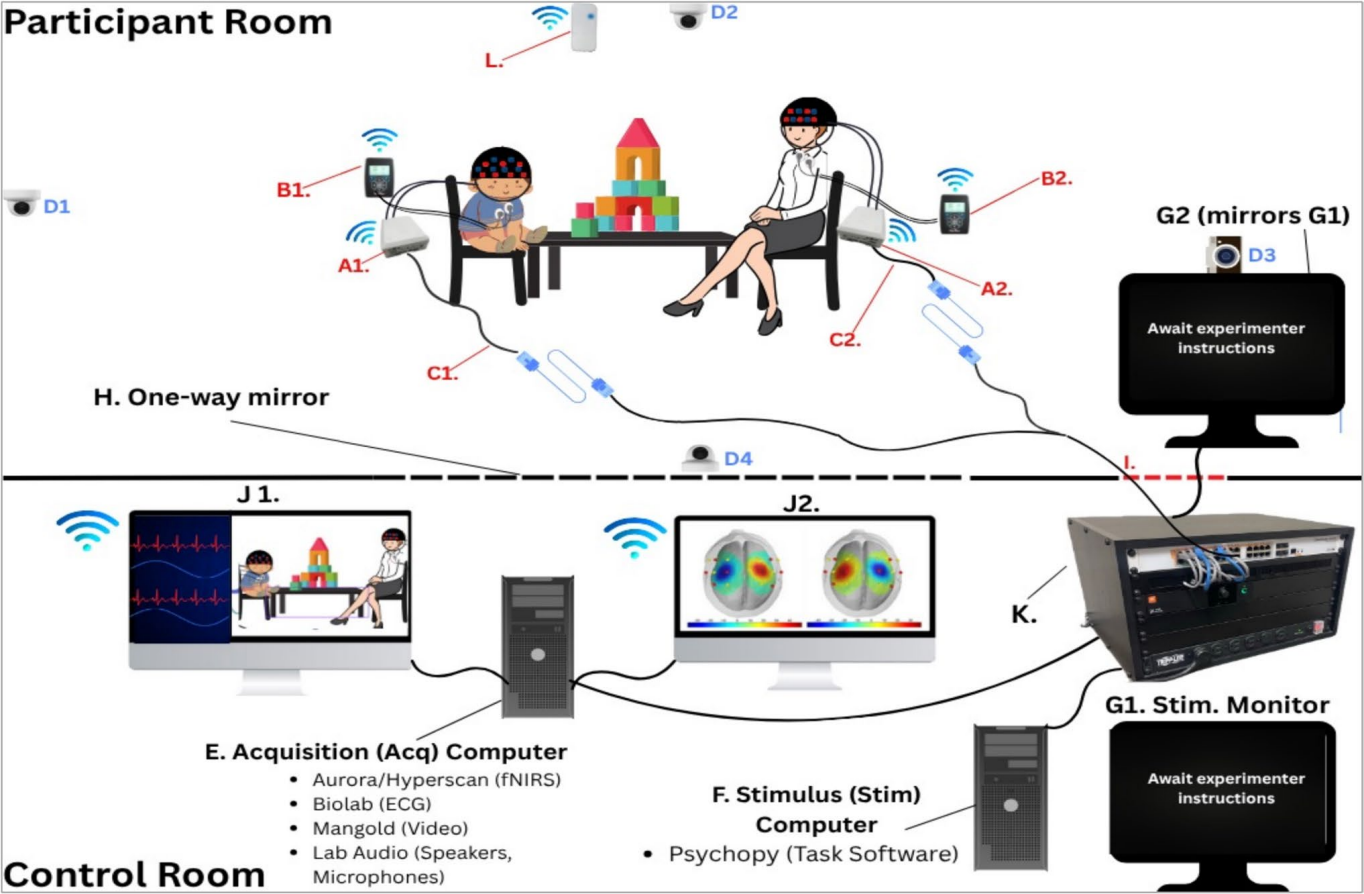
Notes. **A1**- Child fNIRS (Nirsport 2) device (Wi-Fi connection to acquisition computer (E); wired trigger connection (C1) to stimulus Computer (F)). **A2**- Caregiver fNIRS device (Wi-Fi connection to acquisition computer (E); wired trigger connection (C2) to stimulus Computer (F)). **B1**- Child ECG mobile device (Wi-Fi connection to acquisition computer (E)). **B2**- Caregiver ECG device (Wi-Fi connection to acquisition computer (E)). **C1**- fNIRS trigger cable (wires to stimulus computer; G1). **C2**- fNRS trigger cable (wires to stimulus computer; G1). **D (1–4**)- Video Cameras. **E**- Acquisition Computer. **F**- Stimulus Computer. **G1**- Stimulus Monitor 1. **G2**- Stimulus Monitor 2 (Mirrors Stimulus Monitor 2; G1). **H**- One way mirror (separates control and experimenter room). **I**- Sound isolating cable passthrough for trigger cable connection. **J1**- Acquisition Monitor 1. **J2**- Acquisition Monitor 2 (Extend Acquisition Monitor 1; J1). **K**- Mixer Rack. **L**- Mindware Wireless Access Point (L)

Specifically, in our lab, we use one acquisition computer running BioLab, Mangold VideoSyncPro, and Aurora software, and a separate task computer running PsychoPy software. See Fig. 1 for an overview of our setup. BioLab is synchronized with Mangold’s VideoSyncPro to enable frame-accurate video recording, allowing for precise alignment between physiological signals and observed behaviors. To streamline multi-task paradigms, BioLab and Aurora are also integrated with PsychoPy. At the onset of each task, PsychoPy sends a start trigger to BioLab, which initiates ECG recording and automatically starts video recording in Mangold, and marks the task onset in both Aurora and BioLab. An end trigger marks the conclusion of the task, ensuring that both physiological and video recordings are stopped concurrently. This fully synchronized setup is critical for time-locked analysis of physiological and behavioral responses, enabling accurate alignment between stimulus presentation, participant behavior, and physiological reactivity.

Below, we outline the step-by-step process for configuring and synchronizing these systems effectively.

1. **Integrating Mangold VideoSyncPro with BioLab**
  Mangold VideoSyncPro Studio offers precise, multi-camera video recording with frame-accurate timing, and supports integration with physiological recording systems like MindWare’s BioLab. The following steps were followed to enable seamless synchronization:
  1. **Mangold Software Installation and Activation (for a detailed step-by-step guide, refer to MindWare** website here)
    a Install the Mangold Software Manager from the official downloads page.
    b Launch the installer and follow the prompts; restart if required.
    c Activate the VideoSyncPro license via USB dongle or license code provided by MindWare Technologies.
  2. **Configuring cameras and creating a scenario**
    a Set up Axis Cameras through Video Sync Pro and create a synchronized recording scenario. For step-by-step instructions, refer to MindWare’s support documentation here.
  3. **BioLab Configuration for VideoSync Pro**
    For a comprehensive guide on setting up BioLab for integration with Mangold VideoSync Pro, including configuration steps and synchronization settings, refer to the MindWare website here.
    a Install the BioLab software from the official downloads page.
    b In BioLab, navigate to the Audio/Video tab and select Mangold VideoSync Pro as the video source.
    c Enable recording,select the executable path (e.g., C:\Program Files\Mangold VideoSyncPro Studio\Mangold VideoSyncPro Studio.exe), and choose the saved Scenario file.
    d BioLab will then:
      Automatically launch VideoSyncPro.
      Start/stop video recording when BioLab acquisition begins/ends.
      Close VideoSyncPro when exiting the BioLab configuration screen.
      Automatically calculate video delays/offsets during data analysis.
    e Event Synchronization
      i. Event markers are transmitted from BioLab to Mangold
      ii. Both systems reference a common internal clock to align physiological signals and behavioral video frames.
2. **Synchronizing NIRx Aurora/Hyperscan and BioLab using a port replicator.**
  To achieve real-time synchronization between NIRx Aurora/Hyperscan and BioLab systems, we employed a NIRx port replicator, which distributes trigger pulses simultaneously to both systems. For additional details on the NIRx port replicator, refer to the official NIRx website here.
  1. Hardware Setup
    a Connect the port replicator to the task computer via USB.
    b Install necessary device drivers and verify the port address in *Device Manager*.
  2. Physical Connections
    a Connect the replicator’s output to both the NIRx trigger input and BioLab event input using appropriate cables.
    b This ensures that both systems receive identical trigger signals simultaneously.
  3. PsychoPy Port Configuration
    a In PsychoPy, add a Parallel Out component and enter the correct port address assigned to the replicator (e.g., 0x0378 found in Device Manager). See Appendix A-1 and A-3.
3. **Synchronizing Task Events from PsychoPy with Nirx Aurora/Hyperscan and BioLab**
  PsychoPy is used to present task stimuli while sending real-time event triggers to both Hyperscan and BioLab for temporal alignment.
  a Task Design
    Experimental routines are built in PsychoPy to define stimulus onset, offsets, and response events.
  b Sending Event Triggers
    In PsychoPy, use a Parallel Out component (see Appendix A-1) or custom Python code to send triggers at specific timepoints.
    Specify the correct port address for the parallel port communication (see Appendix A-3).
    To enable automatic recording in BioLab upon receiving the start bit from PsychoPy, ensure that “Rising” edge detection is selected in the event detection settings in BioLab:
      Navigate to “Event Detection” in BioLab select the “Rising” option to detect the onset of the trigger pulse.
    The initial trigger value (start bit) on PsychoPy should be > 128 to signal BioLab to automatically begin recording (see Appendix A-2).
    Additional triggers can be inserted to mark critical task events (e.g., stimulus onset, participant response).
      a Example
        i. A sample PsychoPy menu configuration for creating and sending triggers is provided in Appendix A.
        ii. This configuration demonstrates how to send identical triggers to both fNIRS and ECG for precise synchronization.
    Automatic recording cannot be initiated in Aurora; therefore, manually start data acquisition in the Aurora/Hyperscan Control Software to start recording with the NIRx fNIRS system before launching the experimental task in PsychoPy, ensuring the system is ready to receive triggers.
4. **Final Synchronization Validation**
  To confirm full synchronization:
  a Conduct a pilot run using a test scenario.
  b Verify that timestamps across all systems (Aurora/Hyperscan, BioLab, Mangold, and PsychoPy logs) are aligned.
  c Ensure that trigger events are recorded simultaneously and video/audio are in sync with physiological markers.
    **Summary of Synchronization Workflow Steps:**
      1. Initiate BioLab Acquisition:
        Once BioLab is fully configured, clicking *Acquire* will automatically launch Mangold VideoSyncPro and load the designated Scenario for synchronized video capture.
      2. Start fNIRS Recording:
        Manually start data acquisition in the Aurora/Hyperscan Control Software to start recording with the NIRx fNIRS system before beginning the experimental task in PsychoPy task, ensuring the system is ready to receive triggers.
      3. Send Start Bit from PsychoPy:
        When the PsychoPy task begins and reaches the screen associated with the start trigger, it sends a start trigger (e.g., value greater than 128) via the parallel port.
      4. Automated Triggering of BioLab and Mangold:
        Upon receiving the start trigger, BioLab automatically begins ECG acquisition, VideoSyncPro starts synchronized video recording, and Aurora marks the trigger as the start of fNIRS recording (but manually start data acquisition in the Aurora/Hyperscan Control Software before launching the experimental task in PsychoPy), ensuring all modalities are aligned to the same initiation time.
      5. Ongoing Event Marking During Task Execution:
        Throughout the task, PsychoPy continues sending event markers (e.g., for stimulus onset, participant responses). These markers are simultaneously recorded in:
        BioLab (ECG event markers)
        Mangold VideoSyncPro (behavioral video timecodes)
        NIRx Aurora/Hyperscan (fNIRS trigger input)

This structured setup ensures high temporal precision across task stimuli, physiological data, and behavioral recordings. By integrating BioLab, Mangold, Aurora/Hyperscan, and PsychoPy into a unified system, researchers can confidently align multimodal data streams, enhancing the precision and interpretability of studies on cognitive, emotional, and social processes.

## Important considerations for analyzing multimodal synchrony data

While the focus of this paper is on the collection of multimodal data, there are some considerations for analyzing multimodal synchrony data that are worth mentioning. Physiological signals recorded simultaneously during dyadic interaction do not operate on the same exact temporal scale. While traditional approaches would consider this a limitation for properly aligning neurophysiological signals, there are multiple statistical approaches that can handle different timescales across modalities. First, classic time-series approaches can be used across modalities by aligning data streams with different temporal resolutions. For example, analyzing data for both modalities in equal epochs that are long enough to capture changes in both (15–30 s) is one way to analyze neural and RSA synchrony together. Second, there is a growing set of techniques meant to manage this same issue across these and other modalities. For example, approaches such as Granger causality (Xu et al., 2020) and multivariate autoregressive and dynamic multilevel models (Bressler et al., 2022; Hamaker et al., 2018) can be used to analyze time-series data without reducing them to a common timescale. While not much work has used these approaches with multimodal neurophysiological data to date, they are both suitable for managing the complexities of varying timescales across fNIRS, RSA, behavioral, and other types of data. Lastly, synchrony in one modality does not guarantee synchrony in another, and analytic choices should reflect this possibility. The limited existing prior work suggests that detectable coupling may be modality-specific and context-dependent, varying across interaction contexts and brain regions (Nguyen et al., 2021a, 2021b, 2021c; Reindl et al., 2022). Accordingly, synchrony can be analyzed within each modality separately. For fNIRS, researchers could incorporate multi-second temporal lags to accommodate the delayed hemodynamic response likely to occur in caregiver–child interactions, whereas ECG-derived indices such as RSA could use shorter windows and minimal lag adjustment to capture rapid autonomic fluctuations (Cui et al., 2010; Kligfield et al., 2007). Modeling these temporal dynamics independently can further aid in making meaningful cross-modal comparisons as together, these modalities offer complementary views of dyadic coregulation across neural and autonomic systems.

## Data collection

The recommendations presented in this manuscript have been extensively tested in our laboratory. Our ongoing longitudinal study on multimodal hyperscanning examines caregiver–child dynamics during early to middle childhood (ages 3–7) using a battery of dyadic tasks, during two in-person laboratory sessions spaced 1 year apart. The two sessions are similar in type and length of tasks, with some minor modifications. To minimize families’ time in the lab, they can complete consent and caregiver surveys ahead of their visit. Once they come to the lab, we start with a reminder about our in-lab protocol and give the family the opportunity to become more comfortable in the laboratory setting. After all consent and introductions are done, experimenters measure the caregiver and child’s heads and go to setup the caps while the main experimenter continues to build rapport. Once all of this is done, the process of setting up the fNIRS cap and placing ECG electrodes starts.

Before putting on the devices, experimenters demonstrate the ECG stickers and fNIRS caps on a stuffed toy and explain the process in simple, playful language. Children are often more willing to cooperate when they see that the “toy is doing it too.” ECG stickers are introduced as “special stickers,” and demonstrate how they are similar to normal stickers that children are typically fond of. Children are given the option to watch an age-appropriate video while the equipment is being placed on them. In addition, our task designs are embedded within the context of a treasure hunt to maintain motivation and excitement throughout the session. Each task is framed as a game, guided by a trained experimenter, and followed by a small prize to reinforce engagement. We also conduct frequent check-ins of the child’s emotional state and offer a long break during the session to reduce fatigue. Families in our study complete 2–3 individual computer tasks (depending on time point) after which they take their caps off and take a 15–30 min break. After the break, the fNIRS equipment is once again setup and calibrated and the dyads complete a battery of six brief tasks (about 35 min in total). These tasks were selected to elicit different types of dyadic interactive patterns, with activities ranging from co-navigating uncertain situations and solving puzzles to unstructured play. At the end of the session, children received a stuffed animal as a final reward.

Experimental designs that integrate fNIRS hyperscanning, dual ECG data collection, PsychoPy-based tasks, and 360-degree video and audio recording – particularly with children as young as three and their caregivers – present technical and compliance challenges. We have compiled data collection statistics of our ongoing study (*n* = 85, 3 to 7 years old) to better understand these difficulties. A large portion (89%) of participants completed most of the study tasks (at least five out of six dyadic tasks) and demonstrated compliance with the experimental procedures. While children of all ages completed all of the tasks in our paradigm, 89% of the children who did not cooperate with all elements of every task were aged 5 or below, and 56% of them were 3-year-olds. In total, out of 85 participants, nine of them (10.6%) refused to wear one or both physiological recording devices at some point in the study. Outside of these nine participants, two participants had missing hyperscanning data due to technical limitations rather than participant noncompliance. Among the nine participants who were not compliant with the tasks, 44% (4/9) specifically refused to wear the fNIRS cap, 11% (1/9) specifically refused to wear the ECG sticker electrodes, and 44% (4/9) refused both. Some of our technological challenges included ECG electrodes or fNIRS source/detector optodes coming off during tasks, data acquisition or trigger software not opening or working reliably or crashing unexpectedly, and internet instability.

## Strategies for addressing data collection challenges with young children

Addressing or anticipating challenges that commonly occur during data collection with young children while keeping the experience engaging and enjoyable is key for developmental researchers aiming to collect extensive behavioral and biological data across a variety of tasks. Moreover, physiological and neural data collection, especially involving wearable devices like fNIRS caps and ECG electrodes, is typically a novel and intimidating for children, particularly those under the age of five. Our approach to overcoming this challenge is threefold: 1) Ensuring tasks are age -appropriate and enjoyable, 2) Making the technology application as comfortable as possible, and 3) Extensive training for experimenters to properly measure, apply, and operate all technology equipment with children efficiently, while also using child-friendly engagement techniques to promote comfort and cooperation.

To reduce discomfort and resistance, we aimed to keep cap preparation brief and minimize the time children wore the fNIRS cap, making the procedure as child-friendly as possible. Moreover, proper fit and placement of fNIRS cap are crucial not only for comfort but also for ensuring high-quality data. A cap that is too small, not properly assembled, or placed even slightly off can cause discomfort and compromised data quality. NIRx recommends sizing down when selecting cap size (ex. if their head is 55 cm, choose cap 54 rather than 56), while this can improve data quality, our protocol now includes bringing two cap sizes to determine the best fit for the child’s particular head shape. This has resulted in increased child compliance. On occasion, sizing up can sometimes work better for children’s comfort, as they are typically more sensitive to the tightness. We also use low spring pressure settings (0 or 1 for children) on the optode holders to minimize discomfort during fNIRS data collection. Experimenters are trained to accurately measure the head circumference, from inion to nasion, and ear to ear, and select the cap that most closely aligns with the head circumference without compromising comfort. For children, pediatric electrodes with gentle adhesive and low impedance should be used to prevent discomfort and minimize skin irritation.

During the placement and calibration of the fNIR cap and ECG electrodes, children are shown short clips of kids cartoons to reduce anxiety and maintain engagement. Experimenters consistently check in with them and make adjustments as needed to ensure comfort. These procedures have drastically improved the willingness of children to go through with wearing the ECG stickers and fNIRS cap, making the overall experience more comfortable, and contributing to higher-quality data. Finally, we use Goo Gone bandage adhesive remover or a similar adhesive remover to assist with the removal of ECG stickers from the child’s body, as this can make the adhesive-removal process gentler and less distressing for children. We also provide families with the option to remove the ECG stickers at home, along with clear instructions on how to do so safely and comfortably. Some of the at-home removal options they provide include using coconut oil or another similar oil, or taking a warm bath.

## Conclusion

As the field of multimodal hyperscanning continues to grow, there are various yet-to-be-answered questions that it can address. For example, what are the temporal dynamics of neurobiological synchrony during emotional situations? Prior studies using ECG have emphasized the bottom-up arousal processes that become activated during an emotional event, while fNIRS studies have highlighted the recruitment of the prefrontal cortex (PFC) during regulatory attempts. Combining these modalities through ECG-fNIRS multimodal hyperscanning allows for a more comprehensive investigation of the temporal dynamics of emotional processing and regulation in caregiver–child interactions during an emotional event.

The effects of caregiver modeling are another area where multimodal hyperscanning can be useful. Historically, the lack of truly time-synchronized behavioral and neurobiological data streams has limited our ability to make strong inferences between specific instances of caregiver modeling and child behavior during caregiver–child interactions. Multimodal hyperscanning offers a way to do this that allows assessment of the effect of caregiver modeling (assessed via time-synchronized video data) across various levels of neurobiological analysis. Another way in which a multimodal hyperscanning approach, especially one that also includes longitudinal assessments, can be useful is to help better delineate the developmental integration of biological systems. By allowing simultaneous assessment of changes in both neural and physiological systems during the same type of event and tracking these changes over time, researchers can track how these systems co-develop and interact across childhood. While this is not an exhaustive list of questions that can be answered using a multimodal hyperscanning approach, we hope these examples do illuminate the multiple questions that can be addressed with this approach.

While our manuscript focused on fNIRS-ECG-Video integration, as that is our current setup, we acknowledge that there are other integrations that may be of high importance for some laboratories. For example, studies interested in physical activity or visual attention may want to consider modified versions of this setup by integrating accelerometers or eye-tracking to their setup. While a detailed description of these alternative approaches is beyond the scope of this manuscript, we strongly encourage researchers to identify which aspects of caregiver–child interactions are of most importance to their questions and to modify the setup described here to fit those needs.

In the current manuscript, we provide a practical and replicable framework for caregiver–child multimodal hyperscanning that other researchers can adopt and modify for their laboratory needs. By doing this, we hope to broaden the scope of multimodal hyperscanning studies in the field. We strongly believe that increasing the use of multimodal hyperscanning approaches will help advance the field of developmental psychobiology by allowing for a more comprehensive study of the neurobiological mechanisms underlying caregiver–child interactions. While we recognize the numerous limitations that are inherent in multimodal hyperscanning work, especially with young children, collecting truly time-synchronized concurrent ECG and fNIRS hyperscanning data offers a unique opportunity to disentangle bottom-up arousal processes from top-down neural processes, something that is hard to parcel out in studies that only include one modality. We hope this guide serves as a useful starting point for new laboratories to integrate these approaches by demystifying the process.

## Supplementary Information

Below is the link to the electronic supplementary material.

Supplementary file1 (DOCX 112 kb)

## Data Availability

Data sharing is not applicable to this article as no datasets were generated or analyzed during the current study.

## Code availability

Code sharing not applicable to this article.
