# Supplementary material for: Synchronizing brains and hearts: A practical guide for caregiver–child fNIRS-ECG multimodal hyperscanning
Source: Behav Res Methods. 2026 Jun 17;58(7):199. doi: 10.3758/s13428-026-03060-7 (PMC13275591; doi:10.3758/s13428-026-03060-7)
Supplement: Supplementary file 1 — Supplementary file1 (DOCX 112 kb) [file 13428_2026_3060_MOESM1_ESM.docx]

**Appendix A**

Appendix A-1

**
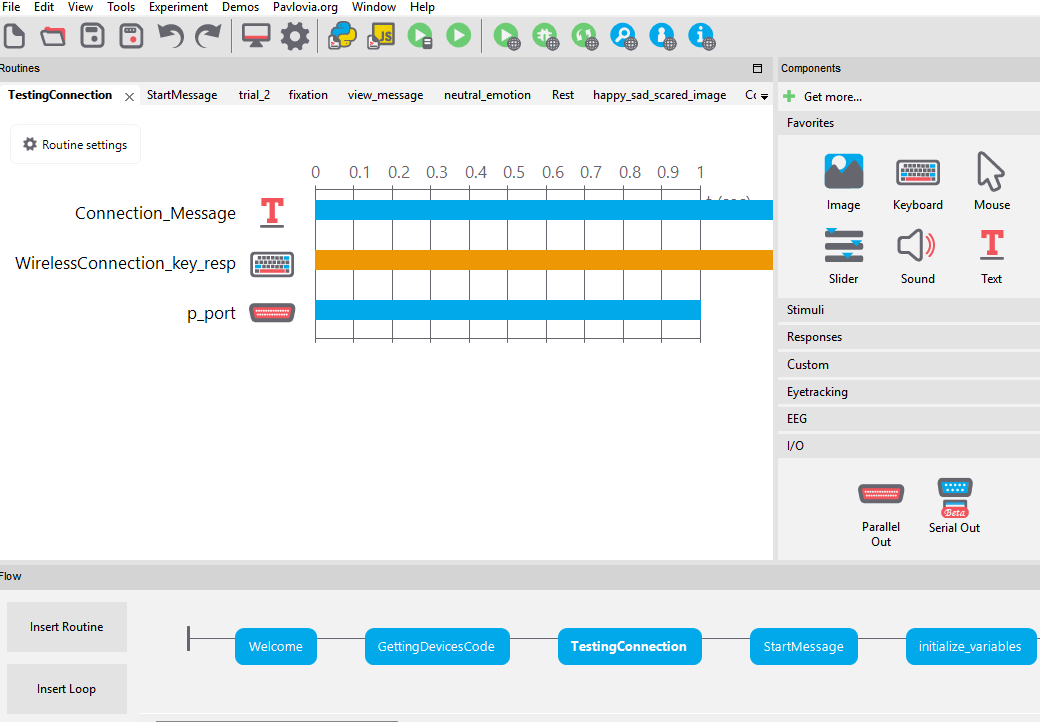
**


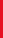

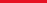

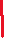

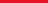


Appendix A-2 Appendix A-3

**
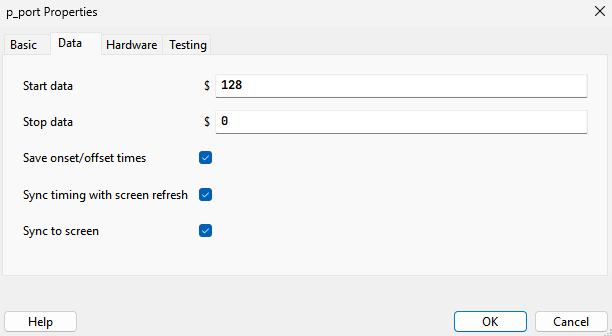

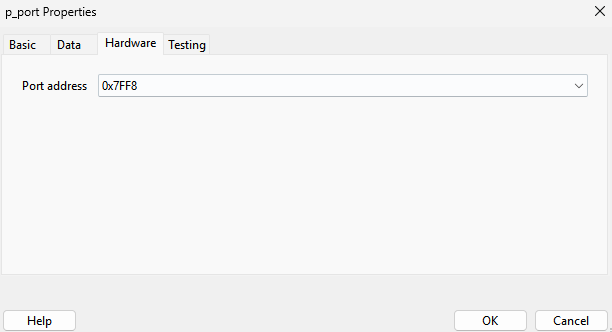
**

**Appendix B**

**NIRx:** The Nirsport 2 emitter diodes utilize high-powered dual LEDs with 32-mW illumination and highly sensitive Avalanche Photodiodes (APD) detectors, detecting as low as 5 fW, to enable optimal spectroscopy. Nirsport caps come in various sizes, accommodating head circumferences from as small as 42-cm caps (typically fitting children as young as 2 or 3 years old) to 60-cm caps. Sources and detectors are secured via spring-top covers or rubber grommets, depending on the participant population and calibration needs. Spring tops come in levels 0 to 4, with increasing spring pressure to improve scalp contact. Rubber grommets provide a secure grip without added pressure, which can be a preferable option for young children or individuals with heightened sensitivity. Short-distance detectors are positioned close to the light sources (~8 mm apart), creating much smaller channels compared to ~30 mm separation used for standard cortical channels. These short-separation channels primarily capture signals from the superficial layers of the scalp and skull, rather than from cortical regions (Gagnon et al., 2014). NirX’s basic acquisition software (Aurora) comes with an additional “hyperscanning” application that enables simultaneous recording from multiple participants. It operates by opening multiple instances of Aurora alongside a separate control interface, allowing for concurrent neural recordings. Individuals can have the same or differing montages, depending on the research needs. The Aurora/Hyperscanning application can detect multiple active Nirsport devices via Wi-Fi or wired connection. The NIRx hyperscanning application enables recording from up to five Nirsport’s devices. This capability allows for real-time data collection of neural activity not only in dyadic-fNIRS data collection but also opens the possibility of studying synchronous neural activity in larger social groups in real time (Nozawa et al., 2016).

**MindWare:** The MindWare platform allows for the wireless, synchronized recording of up to eight participants simultaneously and provides robust capabilities for analyzing sympathetic, parasympathetic, and synchronous physiological activity. While wireless streaming is ideal for laboratory-based recordings, an SD card option is also available for extensive, real-world data collection. The mobile devices themselves are small (4.62” x 3.11” x 1.3, 8.4 oz) and have a clip for easy attachment to participant clothing, chairs, or other surfaces. Furthermore, they feature 24-bit resolution, a sampling rate of 500 Hz, eight recording channels, a built-in accelerometer, a user-friendly electrode interface, and a 24-h battery life. The device is easy to use, with straightforward button navigation, connection to Wi-Fi, and charging. ECG setup is straightforward: color-coded electrode leads correspond to matching color-coded ports on the bottom of the device. Experimenters attach the electrodes to adhesive electrode stickers, place them on the participant’s chest and back, and create a tension loop using tape to minimize movement artifacts. The MindWare platform supports for the analysis of a wide range of physiological measures including: respiratory sinus arrhythmia (RSA), root mean squares successive differences (RRMSSD), pre-ejection period (PEP), total peripheral resistance (TPR), cardiac output (CO), stroke volume (SV), and left ventricle ejection time (LVET). These measures can be analyzed through MindWare’s software programs, such as HRV and IMP.

**Mangold International:** One of its key strengths lies in its robust integration capabilities with physiological recording systems, allowing researchers to conduct comprehensive multimodal analyses (Mangold International GmbH. (n.d)). Specifically, VideoSyncPro Studio is compatible with ECG Mindware systems**,** enabling the accurate alignment of physiological signals with behavioral video data (Mindware Technologies. (n.d.)). While these systems can be integrated within research setups, achieving precise synchronization across platforms often requires custom integration setups. This integration is particularly beneficial in studies that require synchronized behavioral and physiological data. By ensuring frame-by-frame alignment between video and physiological recordings, VideoSyncPro Studio allows researchers to accurately map behavioral responses onto corresponding autonomic or neural processes and also examine moment-to-moment behavioral synchrony between partners. This enhances the temporal accuracy and reliability of behavioral coding, improving the interpretability of psychophysiological data. Moreover, its ability to record and manage multiple synchronized video streams with lip-synchronized audio serves as a critical tool for conducting high-quality, integrative research on human behavior and physiology (Mangold International GmbH. (n.d.)).

**Appendix C**

Although the systems selected for our study were well suited to our specific methodological needs, other hardware and software options may be more appropriate depending on the goals and design of a given study. The examples below highlight companies that currently offer systems relevant to hyperscanning and developmental research; these examples are intended to be illustrative rather than prescriptive.

**Other fNIRS companies/considerations.** For paradigms that require extensive physical activity, such as frequent walking/moving around, OBELAB’s fNIRS system offers lightweight, wireless designs with rapid setup and calibration within a couple of minutes. Additionally, devices such as NIRSIT and NIRSIT Lite have built-in sources and detectors, requiring less of a setup that depends on head-measuring and cap-size selection. Moreover, the OBELAB devices enclose all of the subcomponents in a virtual-reality shaped headset, making them less prone to damage (Ji et al., 2022). The quality of the signal is comparable to NIRX, while having no wires. However, source-detector montages are fixed and limited to the areas of the PFC. Additionally, obtaining dual EEG-FNIRS hyperscanning of the PFC may be more difficult with this design, because the forehead-mounted headband leaves less flexibility for placing EEG electrodes over frontal sites. Nonetheless, fNIRS hyperscanning of the PFC in caregiver-child dyads would be feasible if a child-sized device were purchased in addition to the adult-sized one. Artinis is another company that offers high-quality fNIRS systems that are hyperscanning compatible (St. Clair et al., 2025). Their systems offer muscle tissue oxygenation monitoring, which can be integrated into a hyperscanning design to complement cortical tissue measurements. Short-distance separation channels are available to enhance signal specificity, and the Brite Frontal model, in particular, has fewer wires than the Nirx system and requires minimal setup time.

**Other ECG companies/considerations.** Universiteit Ambulatory Monitoring System (VU-AMS; Netherlands) currently offers a VU-AMS5fs that is used across studies and populations, including young children (Pink et al., 2024). The accompanying software supports a broad suite of autonomic and cardiovascular indices, such as heart rate (HR), interbeat interval (IBI), heart rate variability (HRV), respiratory sinus arrhythmia (RSA), pre-ejection period (PEP), left ventricular ejection time (LVET), respiration rate (RR), stroke volume (SV), cardiac output (CO), skin conductance level (SCL), skin conductance responses (SCRs), and body movement via tri-axial accelerometry noninvasively. Biopac Systems Incorporated (BSI) also provides reliable options for dual ECG recording (Coutinho et al., 2021). BSI provides extensive options to record multiple channels simultaneously and comes with its own analysis software, AcqKnowledge. BSI has an array of products ranging from ECG to EEG to prefrontal fNIRS if researchers are looking to collapse physiological and neural technology into a singular company. Moreover, BSI offers more flexible ECG options, such as a wristwatch model with minimal sticker electrodes, which could be a more suitable, noninvasive option for ECG research with young children in non-laboratory settings.

Other behavioral coding and multimodal observation software considerations. Among its key competitors is the Observer XT by Noldus**,** a leading platform known for strong multimodal data integration capabilities (Noldus Information Technology, n.d.; Zimmerman et al., 2009). Other platforms offer more specialized functionalities. Vicon is well-suited for motion capture and kinematic tracking, making it ideal for biomechanical or movement-focused research (Goldfarb et al., 2021; Vicon Motion Systems, n.d.), but it lacks advanced behavioral coding features. Biopac AcqKnowledge specializes in the acquisition and analysis of physiological signals (e.g., ECG, EEG, and fNIRS) (Sharma et al., 2016) but offers limited tools for behavioral video coding. In contrast, Vosaic provides a cloud-based video analysis solution ideal for collaborative research and education (Vosaic, n.d.; Wada et al., 2022), but it does not support direct physiological data integration. Finally, iMotions offers a comprehensive multimodal research suite, integrating a variety of biosensors (e.g., eye-tracking, EEG, and GSR) with synchronized video and behavioral data (Jmour et al., 2021). Because the platform is modular and often relies on compatible external hardware, it may involve greater setup demands and higher costs depending on the specific configuration.

**Other software companies/considerations**. Several alternative platforms cater to specific experimental needs. **E-Prime** offers a user-friendly graphical interface and is renowned for its high temporal precision (Garaizar et al., 2014). However, its proprietary nature, high licensing cost, and limited scripting flexibility make it less adaptable than PsychoPy for complex experimental designs (Mathôt et al., 2012). Similarly, **Presentation** is known for its **millisecond-accurate timing** and **strong integration with EEG, fMRI, and MEG systems**, but it also requires a paid license and relies on a proprietary scripting language (Neurobehavioral Systems. (n.d.)). **OpenSesame** is another open-source option, featuring a **drag-and-drop experiment builder** combined with Python scripting capabilities (Henninger et al., 2017). It is more accessible to beginners but offers less flexibility than PsychoPy when it comes to implementing complex tasks (Mathôt et al., 2012). **Experiment Builder, developed by SR Research,** is optimized for **eye-tracking experiments**, offering an intuitive drag-and-drop interface. However, it is less versatile for general neuroscience applications beyond eye-tracking (Lin et al., 2022). For MATLAB users, **Psychtoolbox** provides highly precise stimulus control and robust multimodal integration, making it ideal for time-critical psychophysics studies. Nonetheless, its dependence on MABLAH – a proprietary platform – may limit accessibility for researchers seeking fully open-source solutions (Lin et al., 2022). Lastly, **Lab.js** is well suited for **web-based behavioral experiments** and offers a free, open-source environment for online data collection. However, it currently lacks support for real-time synchronization with physiological measures such as EEG, fNIRS, and ECG (Henninger et al., 2021).
